# The current troubled state of the global pathology workforce: a concise review

**DOI:** 10.1186/s13000-024-01590-2

**Published:** 2024-12-21

**Authors:** Elizabeth Walsh, Nicolas M Orsi

**Affiliations:** 1 Women’s Health Research Group, Leeds Institute of Medical Research, St James’s University Hospital, University of Leeds, Wellcome Trust Brenner Building, Beckett Street, Leeds, LS9 7TF UK; 2 Department of Histopathology, St James’s University Hospital, Leeds Teaching Hospitals NHS Trust, Beckett Street, Leeds, LS9 7TF UK

**Keywords:** Pathology, Histopathology, Workforce

## Abstract

The histopathology workforce is a cornerstone of cancer diagnostics and is essential to the delivery of cancer services and patient care. The workforce has been subject to significant pressures over recent years, and this review considers them in the UK and internationally. These pressures include declining pathologist numbers, the increasing age of the workforce, and greater workload volume and complexity. Forecasts of the workforce’s future in numerous countries are also not favourable – although this is not universal. Some in the field suggest that the effects of these pressures are already coming to bear, such as the financial costs of the additional measures needed to maintain clinical services. There is also some evidence of a detrimental impact on service delivery, patient care and pathologists themselves. Various solutions have been considered, including increasing the number of training places, enhancing recruitment, shortening pathology training and establishing additional support roles within pathology departments. A few studies have examined the effect of some of these solutions. However, the broader extent of their implementation and impact, if any, remains to be determined. In this regard, it is critical that future endeavours should focus on gaining a better understanding of the benefits of implemented workforce solutions, as well as obtaining more detailed and updated pathology workforce numbers. With a concentrated effort in these areas, the future of the pathology workforce could become brighter in the face of the increased demands on its services.

## Background

Over recent years, many have been concerned with the apparent decline in the pathology, and especially histopathology, workforce. Given histopathology’s pivotal involvement in most cancer diagnoses, the idea of a workforce shortage is rightly cause for concern, particularly considering the anecdotal increase in demand and complexity of diagnostic services.

This short article aims to provide an overview of the current state of the pathology workforce both in the UK and other countries, with a specific focus on histopathology, where possible. Emphasis is placed on workforce numbers/demographics and the expanding nature of diagnostic work. Possible effects on service provision, patient care and pathology careers will also be addressed, along with workforce predictions and potential solutions to overcome some of the challenges faced by the discipline.

## Number of pathologists

One of the countries with an almost unequivocal shortage of histopathologists is the UK. A workforce census was published by The Royal College of Pathologists (RCPath) in 2018 reporting on the state of the national histopathology workforce [1]. By its estimates, the RCPath found that while there were around 1444 consultant histopathologists in the UK, only 3% of departments surveyed claimed to be adequately staffed to meet clinical demand. Further compounding the problem, 78% of departments reported having vacant consultant posts. Moreover, the authors drew attention to unfilled specialty training posts and the declining number of trainees in the discipline.

In Europe, Märkl et al. reported that Germany had a shortage of pathologists [2]. When they compared figures to another 24 countries in Europe, Germany had the second lowest number of pathologists per inhabitants, only ahead of Poland. They also recognised that there are likely inconsistencies on what the title of ‘pathologist’ represents across countries. In fact, even they did not provide an explicit definition of the ‘pathologists’ they counted in Germany. The authors also surveyed 18 German institutions and most replied that they found coping with workload as either “acceptable” or “difficult”.

When considering pathology in the US, there have been several papers that have estimated the number of pathologists [3, 4]. However, these works provided conflicting figures. A re-evaluation by Robboy et al. investigated these discrepancies and discovered there were distinct differences in how pathologists were counted in the different sources used by both sets of authors [5]. Consequently, published in 2024, Black-Schaffer et al. investigated how best to quantify pathologists in the US and re-evaluated recent histopathologist figures [6]. They suggested that pathologists should be counted as those who have a “pathology specialty or subspecialty designation” recorded in their primary/first and/or secondary/second specialty field within the American Medical Association (AMA) Physician Professional Data™, excluding those with “specific non-pathology designation” in either field. Using this preferred method, the authors counted 17,400 pathologists in 2004, which increased to 20,400 in 2020.

Presently, The Association of American Medical Colleges (AAMC) US Physician Workforce Data Dashboard, which uses the 2023 AMA Physician Professional Data™, reports that in 2022, the number of physicians in pathology was 21,215, with 6.4 pathology physicians per 100,000 population [7]. The pathologist definitions used by the AAMC [8] appear to largely match the recommendations latterly published by Black-Schaffer et al. [6]. For example, whilst the AAMC says that the definitions provided for pathology specialty grouping are “based on the primary specialty code and secondary specialty code”, these codes can be “Other Specialty” or “Unspecified” for either the primary or secondary specialty, suggesting that just one pathology-specific code in either field is enough to be counted [8]. That said, if either of these codes are in the primary specialty, the pathology-specific codes which can populate the secondary specialty are more restricted. Furthermore, these groupings do not appear to include any definite non-pathology specialties across either primary or secondary specialty data fields, suggesting that these physicians are excluded – in line with Black-Schaffer et al.’s advice.

However, Black-Schaffer and co-workers recognise that there are potential limitations to their recommendations [6]. Firstly, they highlight the source of the AMA’s data as a potential issue, suggesting that data are drawn directly from training programmes but also that individual physicians can edit their own data. When looking directly at the AMA’s/AMA’s Credentialing Services’ Description of AMA Physician Professional Data™ for AMA Physician Profiles, it is not obvious which primary source provides data for a physician’s primary or secondary specialty [9]. This document details different “data elements” included in the AMA Physician Professional Data™ database and where “specialty” is mentioned, it appears to fall under multiple different headings. There is the physician-reported “self-designated practice specialty”, the “specialty” or “subspeciality” provided by training data taken from Accreditation Council for Graduate Medical Education-accredited programmes and board certification data from the American Board of Medical Specialties, including subspecialty certificates. When considering US pathologist numbers, some of these data sources may be problematic. For example, if the primary and secondary specialty data originate from training data, they may not accurately reflect a physician’s actual scope of practice. In this regard, Black-Schaffer et al. also discuss the possible impact of pathologists who undertake multiple and/or intercalated fellowships, suggesting that this may affect their AMA Physician Professional Data™ specialty designation [6].

Whilst methods may not perfectly reflect the true pathologist numbers in the US, one way to understand the real-world state of the workforce is from Gross et al.’s assessment of the 2021 College of American Pathologists Practice Leader Survey [10]. The authors made it clear that while they believed that their work could not determine a definite “pathologist shortage”, they nevertheless expressed concern that there may be too few pathologists to meet demand. They also found that just over 46% of practises had 6 full-time equivalent pathologists, with just over 14% reporting 26 or more. Additionally, when considering recruitment, 64.7% of 150 practices were able to fill all their vacancies and just over 26% of posts remained vacant in 2021. This therefore suggests that whilst absolute numbers may have increased, the quantity of pathologists potentially remains inadequate.

In the context of this review, it remains difficult to ascertain specifically the number of histopathologists in the US. This is largely because the AAMC includes pathology specialties such as “Cytopathology”, “Forensic Pathology” and “Medical Microbiology” within some of their pathology specialty grouping [8]. In fact, Black-Schaffer et al. acknowledge that their work does not encompass looking at pathologist numbers within separate subspecialties [6]. To this end, it may be prudent to examine the different subspecialties within pathology to gain a more specific insight into its workforce.

The US is not the only country to report increasing pathologist numbers whilst continuing to experience workforce challenges. A report by Colgan and Geldenhuys showed that the number of Canadian pathologists rose by almost 13% between 1999 and 2009 [11], a trend mirrored by Metter et al.’s reported 20.45% increase in Canadian pathologists from 2007 to 2017 [4]. This has also been seen regionally in Canada, with an investigation from 4 hospitals estimating an increase of 1% between 2011 and 2019 [12]. Despite these growing numbers, all three studies show that the Canadian pathology workforce still faces challenges [4, 11, 12]. Similarly, in Australia and New Zealand, a 2016 report by the Royal College of Pathologists of Australasia (RCPA) also highlighted its concerns for both current and future demand outstripping existing pathologist supply despite absolute pathologist and trainee numbers having increased [13]. The authors state that in 2016, 44.5% and 52.8% of the pathologist workforce comprised of anatomical pathologists in Australia and New Zealand, respectively. This was an increase in anatomical pathologists of 4.3% per year for Australia and an average of 9.8% per year for New Zealand from 2011 to 2016. Trainees in anatomical pathology also increased by 12.3% and by 15.0% in Australia and New Zealand, respectively, over this same period.

Pathologist shortages are also being felt outside the West. For instance, a paper reported a total of 8 pathologists in Cambodia in 2019 [14]. A different study also looked at the number of pathologists in sub-Saharan Africa [15]. From their findings, the most pathologists in one country was 242 in South Africa, but they did not have the most favourable number of pathologists per population. This was reported in Mauritius, with 1 pathologist per 84,133 persons. The most persons per pathologist in the study was Niger, with 1 per 9,264,500 persons while several countries reported no public sector pathologists at all.

Another country in Asia to report on its pathologist numbers is Taiwan, but unlike other countries, their workforce appears to be without the issue of shortages [16]. In this 2011 publication, authors found that there had been an increase in the number of anatomical pathologists from the years 1998 to 2008. During 2004 to 2008 specifically, this translated into an average annual rate increase of just under 5%. They also reported that the number of “board-certified pathologists” during 1995 and 2007 increased on average by 14.6 per year, whilst 2.6 pathologists per year left their license unrenewed. The authors reasoned this demonstrated a “net annual increase” of pathologists. As of May 2009, the number of “board-certified anatomical pathologists” was said to be 361. The researchers also reported that Taiwan had 14.83 anatomical pathologists per million population in 2006. This was comparable to the figures they cited for Japan and higher than those for Korea. The researchers also surveyed newly qualified anatomical pathologists from 2006 to 2008, finding that 86% were consultant pathologists and 4% fellows, and concluded that “the balance of supply and demand seems to be adequate”. Surprisingly, based on some of their observations, the authors also discussed the possibility of an oversupply of pathologists - out of kilter with global trends. These included the rise in first-year residents and the higher proportion of trainees selecting pathology compared to qualified physicians. Whilst to many this may be an unexpected conclusion, the authors’ predictions of their workforce and workload (see later) may also support the idea of oversupply.

## Ageing workforce

The age of the pathology workforce is another factor of concern. The RCPath census found that a quarter of histopathologists were 55 or older and 36% of this group were 60 or older, presaging a “retirement crisis” [1]. The census also said that if all pathologists aged 55 or older were to retire within 5 years, then England would lose 26% of its current workforce. This would be worse in Wales, with a loss of 36%. This predicament is likely exacerbated by unfilled histopathology training posts. A similar scenario was demonstrated in the RCPA report [13]. For the anatomical pathology workforce, 35.5% and 37.5% are 55 or older, and 13.1% and 21.7% are 65 or older, in Australia and New Zealand, respectively. The authors suggested that this could indicate a significant amount of the workforce retiring over the next 10 years or sooner.

Some in the US are similarly concerned about an ageing workforce. When using their recommended method for pathologist quantification and the AMA data covering 2004 to 2020, Black-Schaffer et al. investigated pathologist age [6]. They found that there was an increase in the percentage of pathologists aged between 60 and 69 from 17.9% to 25.0% during the 2004–2020 period, whilst the increase for those younger than 40 was less at 12.7–13.2%. In addition to this, in 2020 they found that over 50% of the pathologist workforce was 50–69 years old. The authors expressed concern that there would not be enough younger pathologists, such as those aged 40 or less or between 40 and 49, to replace older pathologists as they leave the profession. Interestingly, the AAMC US Physician Workforce Data Dashboard shows that in 2022, the percentage of pathologists under 40 was 10.5% [7], suggesting a drop in this age group if following on from Black-Schaffer et al.’s figures [6]. The Dashboard also denotes that those aged 65 and older represented 26.5% of active pathologists in the US in 2022 [7]. These more recent data may in fact indicate a worsening situation for the US.

Some authors do not express aging as a concern for the Taiwanese workforce [16]. Of the aforementioned 361 anatomical pathologists recorded in 2009, 19.1% (69) were over 55 years old and therefore approaching retirement in the next 5–10 years. The authors predicted that pathologist numbers would decrease on average by 7 per year “at most”, as they believe some “officially retired pathologists might move their practices to other institutions”. These data also illustrated that just over 80% of Taiwan’s anatomical pathologists were 55 years or younger in 2009. Whilst appearing more favourable, these figures are not wholly dissimilar to those of countries such as the UK.

## Increasing workload

The work of a histopathologist has changed greatly over recent years, including volume. The RCPath recognised this in their 2018 workforce census, stating that “increasing workload is a particular concern” [1]. They used a Cancer Research UK (CRUK) estimate that predicted that in 2035 new cancer cases would rise by 40% to 514,000 per year. Today, CRUK puts this figure at around 506,000 new cases by 2038–2040, with a 2% increase between 2023 and 2025 and 2039–2040 [17]. The RCPath also states that NHS screening programmes generate increased demand [1].

Additionally, a 2016 CRUK report evaluating UK pathology services claimed that reasons for a rise in pathology services included higher cancer incidence and increased initiatives encouraging early diagnoses [18]. They also attributed the contribution of a rising population, improved cancer survival and other early diagnostic efforts (e.g., a reduced referral threshold) to increasing pathology service demand. For cellular pathology specifically, they cite an increase in histopathology requests of 4.5% annually.

As mentioned, whilst Canada may not be suffering from a decline in pathologist numbers, increasing workload appears to be having an impact. Even though Colgan and Geldenhuys found that the absolute number of pathologists in Canada had increased by 12.9%, so had their estimate of the number of new cancers during 1999 to 2009, which had risen by just over 32% [11]. They calculated that this resulted in a 17.1% increase in pathologist caseload. Using their own figures, Metter et al. also demonstrated that new cancer cases increased by just under 29%, translating into a 7.06% increase in the number of cases per pathologist [4].

In terms of the US’s current pathology workload, a study by Arvisais-Anhalt, Araj and Park investigated the level of pathologist involvement with Medicare services between 2012 and 2017 [19]. They reported an increase of just under 8% for pathology services provided, as well as just over a 4% increase in the number of pathology services performed per pathologist. In relation to Medicare beneficiaries, it was noted that the number served by one pathologist went from 1,382 to 1,489. Whilst authors also saw the number of pathologists providing Medicare Part B services increase by 3.7% between 2013 and 2017, they recognised that their findings still suggested an increased workload for pathologists in this setting. A similar assessment was made in the RCPA report where figures of billed services from their Medical Benefits Schedule from 2011 to 2016 showed an average increase of 5.6% annually in tissue pathology [13]. Whilst these works provide an insight into the pathology workload of their respective countries, their findings are limited. The RCPA report acknowledges that they have not considered data from public sector hospitals [13] and, similarly, Arvisais-Anhalt, Araj and Park recognise that their work does not translate to the private sector [19].

Unlike other countries, some investigators in Taiwan do not predict a worrying increase in workload [16]. The authors presumed that the number of specimens that pathologists would receive in future “increase only slightly” largely as a result of the aging population.

## Increasing complexity of work

Another contributor to the pressure on the pathology workforce is the increasing amount and complexity of work needed per case, as is recognised in the CRUK report [18]. The authors reason that this is due to an increase in biopsies per patient, greater resection specimen sampling (to meet multi-disciplinary team and RCPath reporting standards), the introduction of new, additional testing and increased time to visually assess early malignancies. The report discussed data from 10 laboratories that showed that from 2009 to 2010 to 2014–2015, the average annual increase of slides and blocks was 4.2% and 3.5%, respectively, outweighing a 3.3% annual increase in requests. A second source cited showed that across the UK there had also been a 20% increase in immunohistochemistry tests per request over 2007–2008 to 2014–2015, despite the average number of slides per case remaining constant. Overall, the authors reason that these findings suggest a higher level of case complexity.

A paper by Warth et al. also presented findings regarding pathology workload from a single institution in Germany [20]. They reported that the number of slides per case increased by over 60% between 2006 and 2014. They also claimed that between 2007 and 2014, immunohistochemistry slides per case nearly doubled, and the proportion for molecular testing more than tripled. However, no absolute numbers are provided and the figures included in the manuscript suggest that the use of immunohistochemistry per case has not doubled, but rather increased by almost 50%.

Another area that the RCPath recognise as potentially contributing to the rising complexity of work and demand for pathologists’ time is genomics and molecular testing [1]. An Expert Group in the RCPA’s report also stated that genetic testing, case complexity and precision medicine are considered as “high” “demand drivers” for “service growth” in Australian anatomical pathology [13]. A slightly different perspective around the increase in demand for molecular pathology and further advances in pathology is seen in Hsu, Jung and Chuang’s 2011 publication from Taiwan [16]. They portray these as an opportunity for their workforce rather than an impending problem, by creating new roles that could “ease the pressure of the rapid increase” in anatomical pathologists.

An increasing workload per case has also been seen in reports that have witnessed a decrease in absolute case numbers. The previously referenced study profiling regional Canadian hospitals between 2011 and 2019 showed a 6% decrease in case numbers paralleled by a rise in the work performed for each case [12]. They found that, when analysing data from surgical, non-gynaecological cytology and gynaecological cytology reports over 4 different hospitals, the number of blocks per year increased by 20%. They also found a 19% increase in the length of diagnosis, microscopic and synoptic case report sections. Finally, they employed two workload models that both revealed a 21% and 23% increase in workload units. Thus, despite a decrease in case numbers, the authors still concluded that pathology workload had increased without a matched increase in the workforce.

## Effects on service provision, patient care and pathologists

Given the number of challenges facing the pathology workforce, it is unsurprising that there is concern regarding service delivery, pathologists and patients. The RCPath workforce census reports that UK departments are already being affected and employing several remedial measures [1]. Of the departments surveyed, half reported utilising locums, and 45% were sending work outside the department. These measures have a financial impact. From respondent information, the RCPath extrapolated that the use of locums and outsourcing of work cost £17 million and £10 million per year, respectively, UK-wide.

The RCPath was also concerned that the forecasted decrease in the histopathology workforce could “put clinical services in jeopardy” [1]. This sentiment was echoed by the CRUK report which expressed that without intervention, waiting times would probably rise due processing and reporting delays [18]. This could translate into delays in diagnoses and treatment. They present data from NHS England that show that from 2010–2011 to 2015–2016, patients waiting over 6 weeks for any pathology diagnosis had increased by around 17% a year. The most recent data therein showed that of the 1650 waiting for a pathology diagnosis, approximately 42% were awaiting a histopathology diagnosis. A study by Wolfe et al. offers further evidence of the impact on patient care [21]. From their survey of histopathologists, delays to dermatopathology reporting had contributed to complaints or serious incidents for 25% of respondents.

The impact on pathologists themselves is something that the CRUK report also considers [18]. It explains that, for cellular pathologists, areas of their career such as teaching, leadership and research have been naturally deprioritised to keep up with demand. Authors have said that the lack of research and academics within pathology is “troubling” and feel this will have a detrimental impact on cancer research and slow progress. This is a sentiment echoed by a survey carried out by Smith et al. in the US [22]. Academic pathologists that responded to their survey appear to have reported that higher amounts of patient work have not allowed time for “academic and educational work”.

Moreover, it is not just pathologist careers which are suffering. Studies concerning pathologist burnout have been published by groups in the US, Canada, Switzerland and Turkey [22–26]. It is also an issue affecting pathology trainees [27]. Worryingly, over 50% of 438 pathologists surveyed in a 2023 US-based study reported burnout, with authors calculating that, overall, “control over workload” ranked first as a stressor for this cohort [22]. An additional US publication from 2020 found that 71.4% of surveyed pathologists had experienced feeling burnout [23]. Similarly, this study found that “increased volume or case workload” was the most common factor attributed to burnout, cited by 42.4% of 231 responses. In fact, authors found a significant relationship between workload and burnout where, of those who reported feeling “moderately overwhelmed by workload or very overwhelmed by workload”, just over 63% were also currently suffering burnout.

Similar rates of burnout have been reported in other countries. In Canada, a study revealed that 58.9% of surveyed anatomic pathologists were found to be “burned out” [24]. Again, workload was demonstrated to be the “number one stressor”, featuring in 35% of responses when participants were asked about the “most stressful” parts of their work. Studies from Europe have slightly more varied findings. Results from Turkey followed the same trend, with 45% of pathologists recording having felt “burnt out” [26]. However, a study in Switzerland including both consultant and resident pathologists reported that just 8.6% of respondents answered “Yes” when asked about burnout [25]. While these authors describe burnout as “not rare among study participants”, their rates are comparatively low.

Experiencing burnout may additionally impact on pathologists’ careers. For example, the survey of US pathologists conducted by Garcia et al. found that among those where burnout was a “current issue”, some contemplated moving to a different laboratory, taking retirement or a complete career change (41.6%, 34.4%, 31.2%, respectively) [23]. Furthermore, the potential effect on patient care has also been considered. From the Canadian study, Keith voices concern surrounding burnout in the of context of patient safety and medical errors [24].

These surveys highlight that burnout is an endemic problem affecting our workforce and in need of urgent attention.

## Workforce predictions

Attempts have also been made to predict and quantify how the pathology workforce may look in the future and identify what challenges may lie ahead. The RCPA report included workforce modelling that predicted how many trainees and new fellows were needed in Australia and New Zealand by 2030 [13]. Models were based on two different drivers – either service demand or workforce demand. Within anatomical pathology, the authors found that for Australia, 40 new trainees and 37 new fellows would be required when looking at workforce demand, and 45 new trainees and 41 new fellows when considering service demand. In New Zealand, both service and workforce demand models required an additional three trainees and three fellows by 2030.

In the US, extensive studies have previously examined the potential outlook of the pathology workforce and provided workforce projections [3, 28]. However, these studies were completed before Black-Schaffer et al. published their more robust method quantifying pathologist numbers [6]. As a result, to better understand the future of the American workforce, new modelling should be carried out. Presently, a useful insight comes from Gross et al., who as well as analysing the 2021 College of American Pathologists Practice Leader Survey, also completed a straight-line extrapolation to look at pathologist demand for all US practices [10]. They found that for their lower estimate (1000 practices), 700 pathologists a year could be needed soon, while for their higher estimate (1200 practices), the number may be as high as 840 per year.

As previously discussed, Taiwan appears to be anomalous amongst other countries concerning their pathology workforce and this includes their workforce predictions. Using their results, Hsu, Jung and Chuang predict that there will be 23 newly qualified anatomical pathologist in 2009–2012 [16]. From this they forecast that in 2010, and subsequently in 2015, the number of pathologists will be 400 and 500, respectively. Extrapolations based on survey responses also predicted that annual pathologist vacancies would fall short of the anticipated number of newly board-certified anatomical pathologists.

## Potential solutions

Whilst identifying the issues facing the pathology workforce, many have also discussed potential solutions. One area of focus is pathology training, with the RCPath census stating that “more funded training places” were needed [1]. Additionally, they feel “golden hellos” are also necessary when trying to attract trainees “in hard to recruit areas” [1]. From a German perspective, whilst not providing a figure to allow for quantification, Warth et al. also expressed that future recruitment depended on the existence of “a sufficient number” of training posts [20]. Furthermore, part of a series looking at pathology and laboratory medicine in low- and middle-income countries also identified “increasing the number and quality of pathology graduate teaching programmes” as a way of tackling staff shortages [29].

It has additionally been suggested that increased efforts are needed to attract people to the profession. At undergraduate level, in Germany, both Märkl et al. and Warth et al. considered promoting pathology within medical schools to aid recruitment [2, 20], an idea echoed by Sayed et al. for low- and middle-income countries [29]. Moreover, some studies looking specifically at why individuals do not choose, or do not plan to choose, pathology as a career demonstrated that medical school pathology plays a major role. Surveys from Canada and Australia involving postgraduates and medical students, junior doctors and pathologists, respectively, reported that lack of exposure to/insufficient experience of pathology during medical school as one of the main reasons people did not/would not choose the specialty as a career, only second to wanting more patient contact [30, 31]. Interestingly, another Canadian study using a focus group of senior medical students found that the “most important antipathology influence” for them was that “pathology is clinically invisible” [32]. Students appeared to report having a lack of experience and understanding of pathology during their clinical years. From the results of the Australian survey, authors suggested “active interventions” to increase medical student (as well as prevocational doctors) exposure to pathology is needed to address potential workforce deficiencies [31]. Similarly, one of the Canadian studies suggested educating students about a pathology career in their preclinical years and using clinical clerkships for students to provide more clinical exposure to pathology [32]. The other proposes that pathology departments could “be more aggressive about clerkship exposures to pathology” in early student years to help influence speciality selection [30].

Along those lines, the University of Wisconsin Department of Pathology and Laboratory Medicine in the US currently runs the Angevine Fellowship scheme [33]. This gives medical students who have completed their first year of medical school an internship for 10 weeks. Giving an idea of the fellowship’s effectiveness, Brooks et al. found that 40% of the previous fellows had gone on to be matched in a pathology specialty residency programme [34]. A study from Schukow et al. also described a scheme for US and Canadian high school students: The Pathology Outreach Program [35]. This hopes to stem predicted pathologist shortages by educating students about pathology and laboratory medicine through interactive sessions. Whilst the authors realised that it was premature to establish if the programme was helping to increase entry into pathology, they showed that 96% of the students reported an increase in “their understanding about pathologists’ role in patient care”.

In the UK, the CRUK report also suggested that those medical schools without pathology in their curriculum could consider including it [18]. They even suggested that it may be valuable signposting science undergraduates to postgraduate medicine with a view to eventually joining the pathology specialty. Sayed et al. similarly considered that pathologists needed to be “more visible” within medical schools [29]. Two of the ways they suggested that this could be done is by pathologists becoming involved in the design of curriculum and taking on the role of course directors. A similar sentiment has come from the Australian survey from Fielder et al. and the Canadian survey from Ford [30, 31]. Fielder et al. also proposed that pathologists should be included when designing the curriculum for medical schools and that those “who are good and engaging teachers”, including residents, would help improve the “profile of pathology” [31]. Ford also suggested that having “more positive, enthusiastic pathologists” interact with medical students could help recruitment to the specialty [30].

For trainee doctors, the RCPath workforce census discussed making more pathology places available during foundation training and offering “tasters” in the specialty [1]. Fielder et al. also proposed that prevocational doctors should be exposed more to pathology via rotations [31], a sentiment echoed by CRUK [18]. The latter also proposed that it could be worth considering lessening the level of clinical experience needed to become a pathologist to help with a more “rapid introduction” to pathology.

Some have also proposed changes to training programmes that may help with the workforce. In the US, Gross et al. have considered that trainees themselves opting to shorten their fellowship years could address pathologist shortages [10]. They also said that, anecdotally, more trainees are not completing a second year. The authors recognised that this is a temporary solution with the real impact of this alleged trainee choice still not currently fully understood. When discussing the state of the German workforce, Märkl and co-workers went one step further and suggested that the introduction of a “federal level” training programme and decreasing the length of training could be beneficial [2]. The utilisation of pathology trainees to help with workloads has also been considered. In the UK, the CRUK report recognised that senior trainees could report without direct supervision, potentially lessening consultant workloads [18]. In this respect, the RCPath has published guidance regarding trainee independent reporting [36].

As well as focussing on recruitment and trainee-based solutions, CRUK’s report also provided recommendations regarding pathologist retirement [18]. They suggested that recruiting internationally could help with “acute shortages” secondary to retirements and that efforts should be made to encourage consultants close to retirement to remain in post. This includes introducing measures like reporting at home and flexible working. Authors in the US have also contemplated international recruitment. Ramos et al. suggested that alleviating the visa difficulties met by non-US international medical graduates in gaining residency posts could hopefully help with shortages across the American workforce, in which they include pathology [37].

Solutions are also being sought that do not rely solely upon pathologists. Both the RCPath and CRUK have looked at alternative roles within pathology departments that could prove useful [1, 18]. Training scientists to enable them to cut up and report cases, and work beside histopathologists was something the RCPath census said more funds should be allocated to [1]. They also expressed the desire to develop “advanced clinical practitioner apprenticeships”. This is another solution on which the RCPath and CRUK are aligned, with the latter having included recommendations that specimen dissection and reporting should be carried out by biomedical scientists where appropriate [18]. They also advocated the role of clinical scientists in pathology departments. In a recent joint publication from the RCPath and the Institute of Biomedical Science, 16 biomedical scientists had finished the RCPath and Institute of Biomedical Science reporting qualification, and 72 were actively training [38]. When considering the potential time saving effect of this intervention, a month-long study in 2004 based out of laboratory in Scotland, found that using biomedical scientists for specimen dissection saved around 16 h of consultant time monthly [39]. Similarly, from results generated in a year-long, retrospective study in Italy, researchers extrapolated that pathologists’ assistants’ specimen examinations saved the equivalent of around 556 cases per year-worth of work for each pathologist [40].

Elsewhere, in Germany, Märkl et al. and Warth et al. both acknowledged that the deputation of certain pathologist tasks to other appropriate staff could be helpful [2, 20]. Interestingly, in the US, Kenney and Broda published details of their pathologists’ assistant training programme at Duke University some time ago back in 1974 [41]. In more recent years, many studies have been conducted examining the roles of pathologist assistants in the US [42–45]. The American Association of Pathologists’ Assistants states that pathologists’ assistants are “trained to provide accurate and timely processing of a variety of laboratory specimens” [46]. They detail that this includes “macroscopic examination and evaluation of all surgical pathological specimens”, as well as taking part and assisting in post-mortems.

Another area that the RCPath and CRUK have similarly implicated in improving the pathology workforce is digital pathology [1, 18]. The RCPath stated that investment to allow for the wider deployment of digital pathology is needed and this should allow “staff to work more efficiently, flexibly and remotely” [1]. They have also produced “best practice” guidelines for its implementation [47]. The need for this investment into digital pathology is mirrored by the CRUK report, where it is included in their recommendations for future-proofing pathology [18]. They support the use of digital pathology to improve efficiency by assessing histology slides “on-screen” and “sharing results”. They also credit it for potentially playing a part in helping with the retention of those planning on retiring by enabling home working. Märkl et al. have additionally identified that “digitalization” could prove very valuable in alleviating pathologist workload [2]. Returning to Metter et al.’s work, they also included “digital imaging of slides” as a factor that they feel will continue to promote greater efficiency by enabling slides to be reviewed remotely [4]. Moreover, artificial intelligence has also been touted as a solution to provide pathologists with improved efficiency [48]. Additionally, it has been highlighted by some as a potential aid to help with workforce shortages [49], with some identifying a great need for artificial intelligence technologies that could increase the amount of diagnostic work managed by pathologists [50]. Others have also voiced that as departments start to digitise against this background of present and projected workforce shortages, computer-aided diagnosis “will almost certainly become the real focus” of the upcoming years of research conducted in digital pathology [51].

## Conclusions

In conclusion, it is clear the pathology workforce, including histopathology, is facing numerous, worrying challenges. As workload, clinical and service demands continue to increase, the existing workforce is simply inadequate to support it. Future projections also demonstrate this situation is only likely to worsen.

While a number of proposed solutions have been discussed, given the differences in healthcare systems internationally, it is unlikely that ‘one size fits all’. For example, it is the opinion of some in the US that the utilisation of alternative pathology staff promotes a “scope of practice creep”, and could affect the success and outlook of the pathology profession [52]. Work needs to be done in individual countries to better understand their own workforce landscape and to propose what solutions would fit best. There is also a need to establish what interventions: (i) have been implemented and (ii) have had any impact. In this vein, an update to the RCPath workforce census is warranted to understand how the histopathology workforce in the UK has progressed over recent years, whether any interventions have been employed and the difference, if any, these have made. A similar update is needed on the very important work carried out by CRUK previously. With up-to-date, accurate workforce data, timely implementation of correct solutions and engagement with technological advances, the pathology crisis can be faced head on, and the tide may start to turn.

## Data Availability

No datasets were generated or analysed during the current study.

## Abbreviations

RCPath: Royal College of Pathologists
AAMC: Association of American Medical Colleges
AMA: American Medical Association
RCPA: Royal College of Pathologists of Australasia
CRUK: Cancer Research UK
